# Supplementary material for: Molecular complexity of the major urinary protein system of the Norway rat, Rattus norvegicus
Source: Sci Rep. 2019 Jul 24;9:10757. doi: 10.1038/s41598-019-46950-x (PMC6656916; doi:10.1038/s41598-019-46950-x)
Supplement: Supplementary file 1 — Supplementary material [file 41598_2019_46950_MOESM1_ESM.pdf]

Supplementary material

# Molecular complexity of the major urinary protein system of the Norway rat, *Rattus norvegicus*

Guadalupe Gómez-Baena[1], Stuart D. Armstrong[1], Josiah O. Halstead[2], Mark Prescott[1], Sarah A. Roberts[2], Lynn McLean[1], Jonathan M. Mudge[3], Jane L. Hurst[2], Robert J. Beynon[1¶]

[1]Centre for Proteome Research, Institute of Integrative Biology, University of Liverpool, Crown Street, L697ZB, Liverpool, United Kingdom

[2]Mammalian Behaviour and Evolution Group, University of Liverpool, Leahurst Campus, Neston, United Kingdom

[3]EMBL-EBI, Wellcome Genome Campus, Hinxton, Cambridgeshire, CB10 1SD, United Kingdom

**Supplementary Figure 1|** Workflow followed to analyze rat urine samples.

**Supplementary Figure 2|** ESI-MS intact mass deconvoluted spectra from individual Wistar Han and Brown Norway males.

**Supplementary Figure 3|** ESI-MS analysis of female rat urine.
Deconvolution of the spectrum estimates two masses of about 11 kDa (11065 and 11450 Da) likely corresponding to the rat urinary proteins 1 and 2.

**Supplementary Figure 4|** Sequencing of MUP isoforms from Wistar Han males by native electrophoresis of urine followed by in-gel LysC digestion and analysis by PMF and LC-MS/MS.
Peptide maps show sequence coverage. Red boxes show unique peptides for the isoform and blue boxes show common peptides to several MUP isoforms.

**Supplementary Figure 5|** Sequencing of MUP isoforms from Brown Norway males by native electrophoresis of urine followed by in-gel LysC digestion and analysis by PMF and LC-MS/MS.
Peptide maps show sequence coverage. Red boxes show unique peptides for the isoform and blue boxes show common peptides to several MUP isoforms.

**Supplementary Figure 6|** Sequencing of MUP isoforms from wild caught males by native electrophoresis of urine followed by in-gel LysC digestion and analysis by PMF and LC-MS/MS.

Peptide maps show sequence coverage. Red boxes show unique peptides for the isoform and blue boxes show common peptides to several MUP isoforms.

**Supplementary Figure 7|** Sequencing of MUP isoforms from Wistar Han males by ion exchange chromatography fractionation followed by LysC digestion and analysis by LC-MS/MS.

ESI-MS intact mass deconvoluted spectra from individual Wistar Han are shown for each fraction.

**Supplementary Figure 8|** Sequencing of MUP isoforms from Brown Norway males by ion exchange chromatography fractionation followed by LysC digestion and analysis by LC-MS/MS.

ESI-MS intact mass deconvoluted spectra from individual Brown Norway are shown for each fraction.

**Supplementary Figure 9|** Network representation of a comparison of the expected trypsin peptides from protein sequences of the predicted rat MUP isoforms, highlighting unique peptides for each isoform. Peptide mapper [60] was used to perform in-silico digestion of protein sequences and network was built using Cytoscape [61].

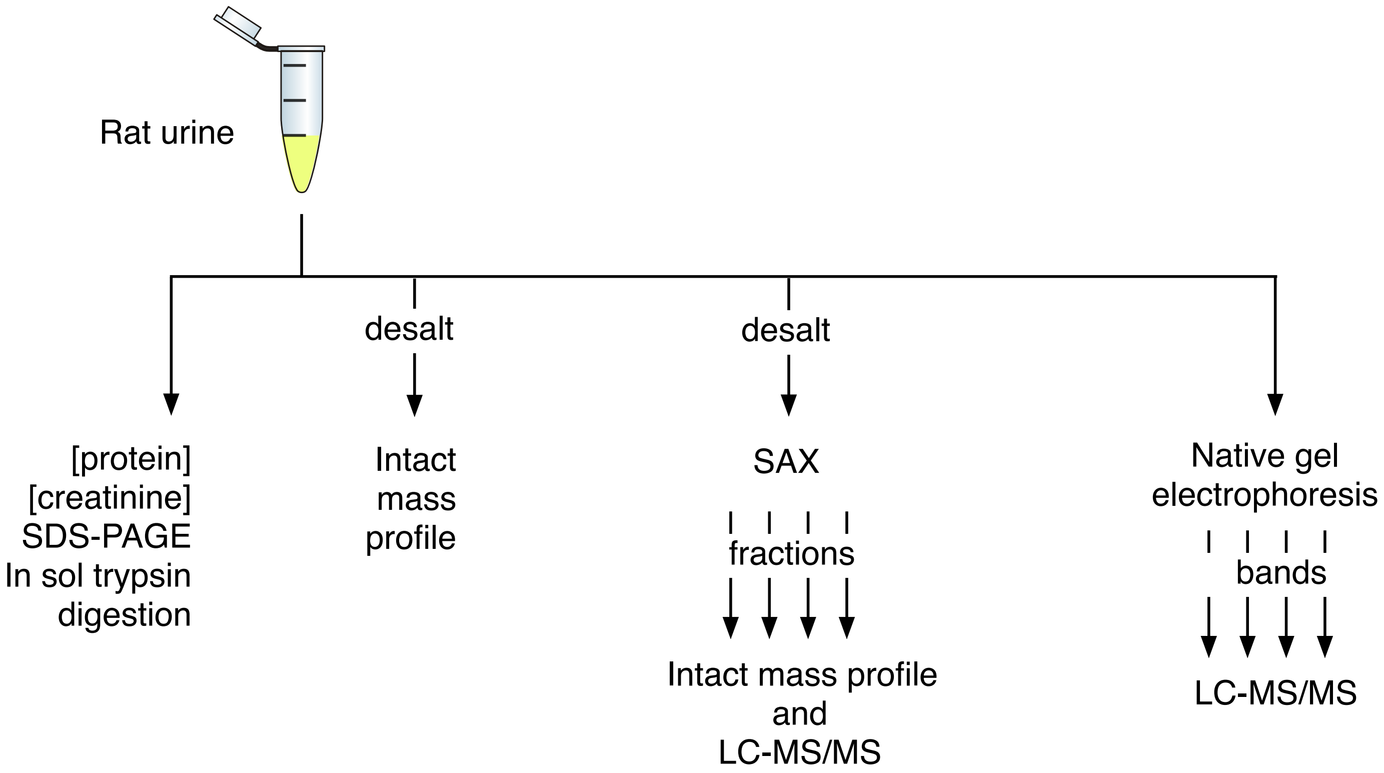


**Supplementary Figure 1: Diagram of the workflow followed to analyse the urine samples**

Supplementary Figure 2: Individual intact mass spectra from Wistar Han and Brown Norway animals.

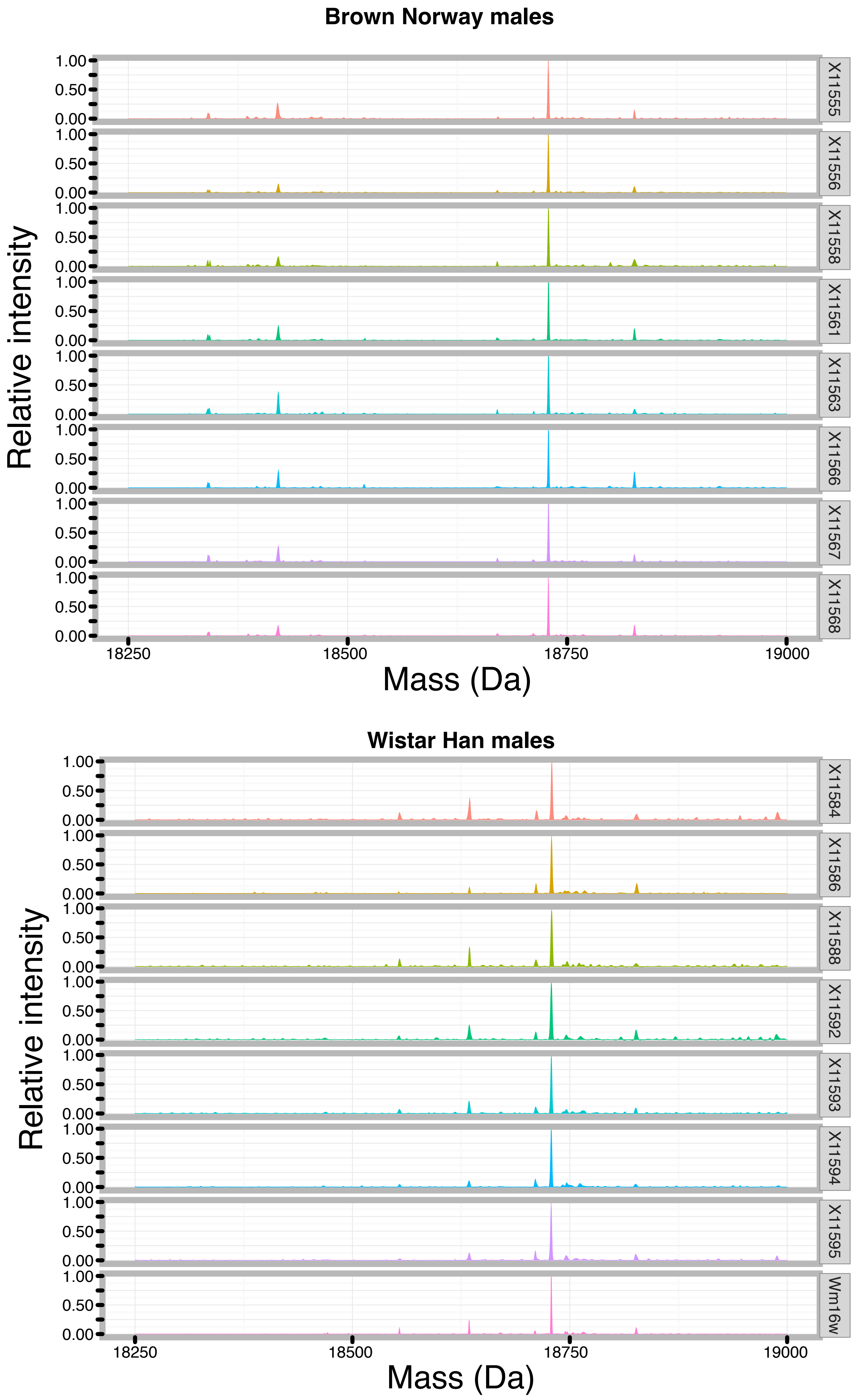

Supplementary Figure 3. ESI-MS analysis of female rat urine. Deconvolution of the spectrum estimates two masses of about 11 kDa (11077 and 11408 Da) likely corresponding to the rat urinary proteins 1 and 2 including N-linked glycosylation.

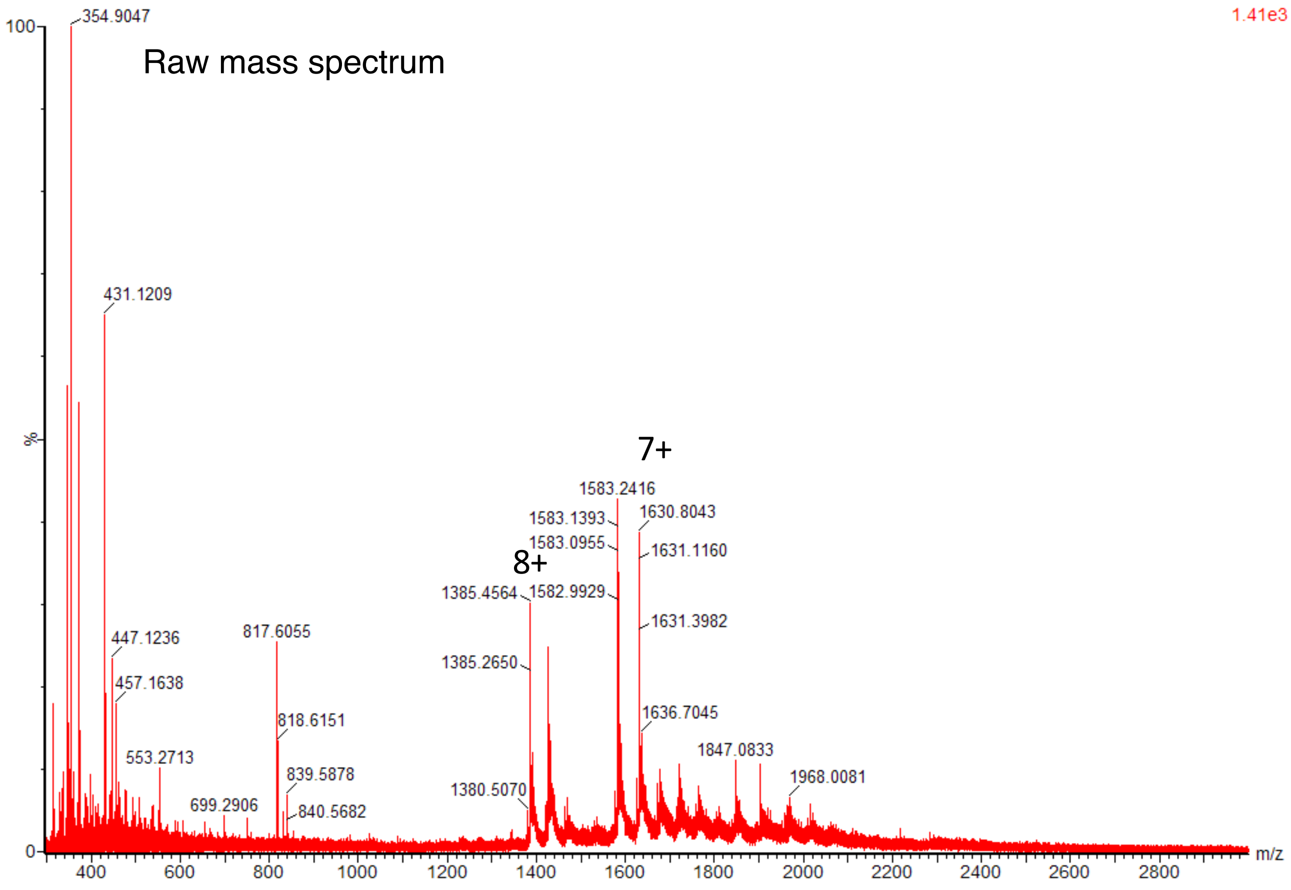


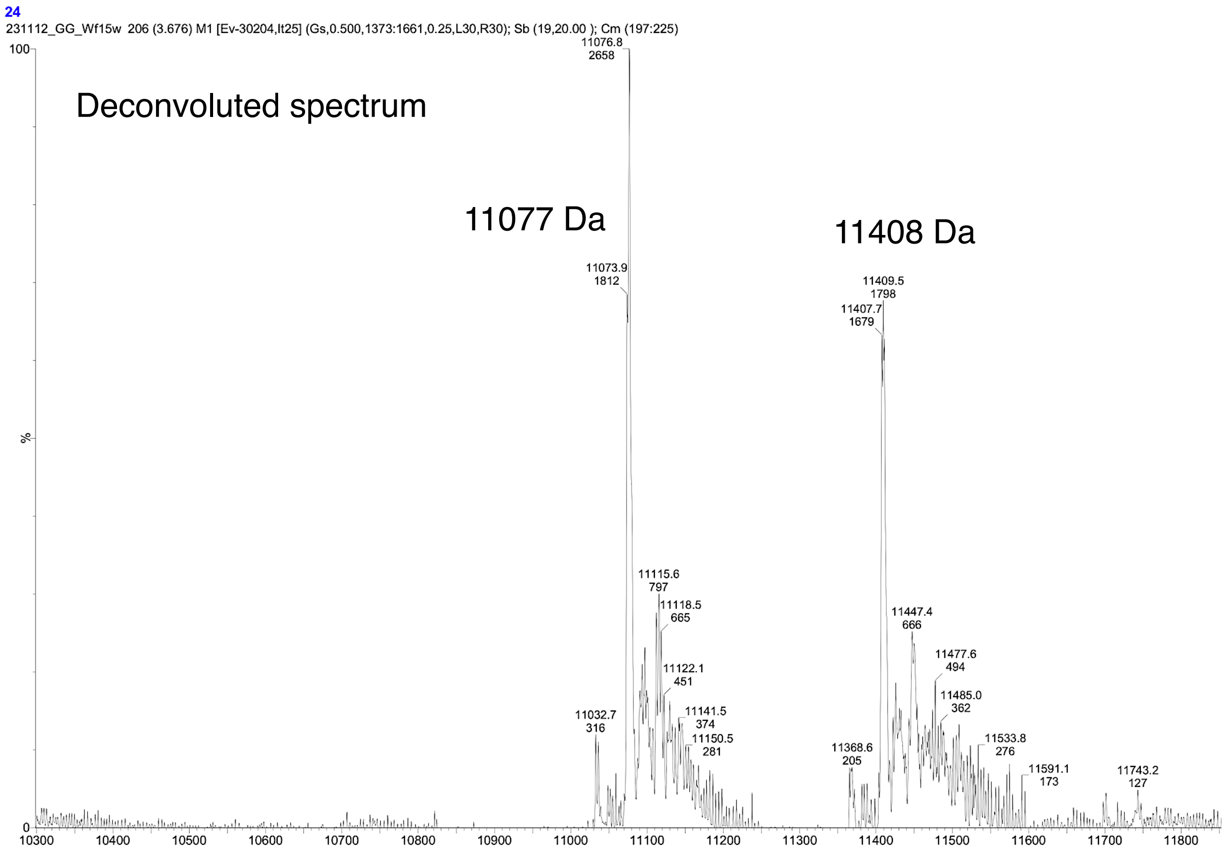

Suppplementary Figure 4. In-gel analysis of urinary MUPs from Wistar Han male rats.

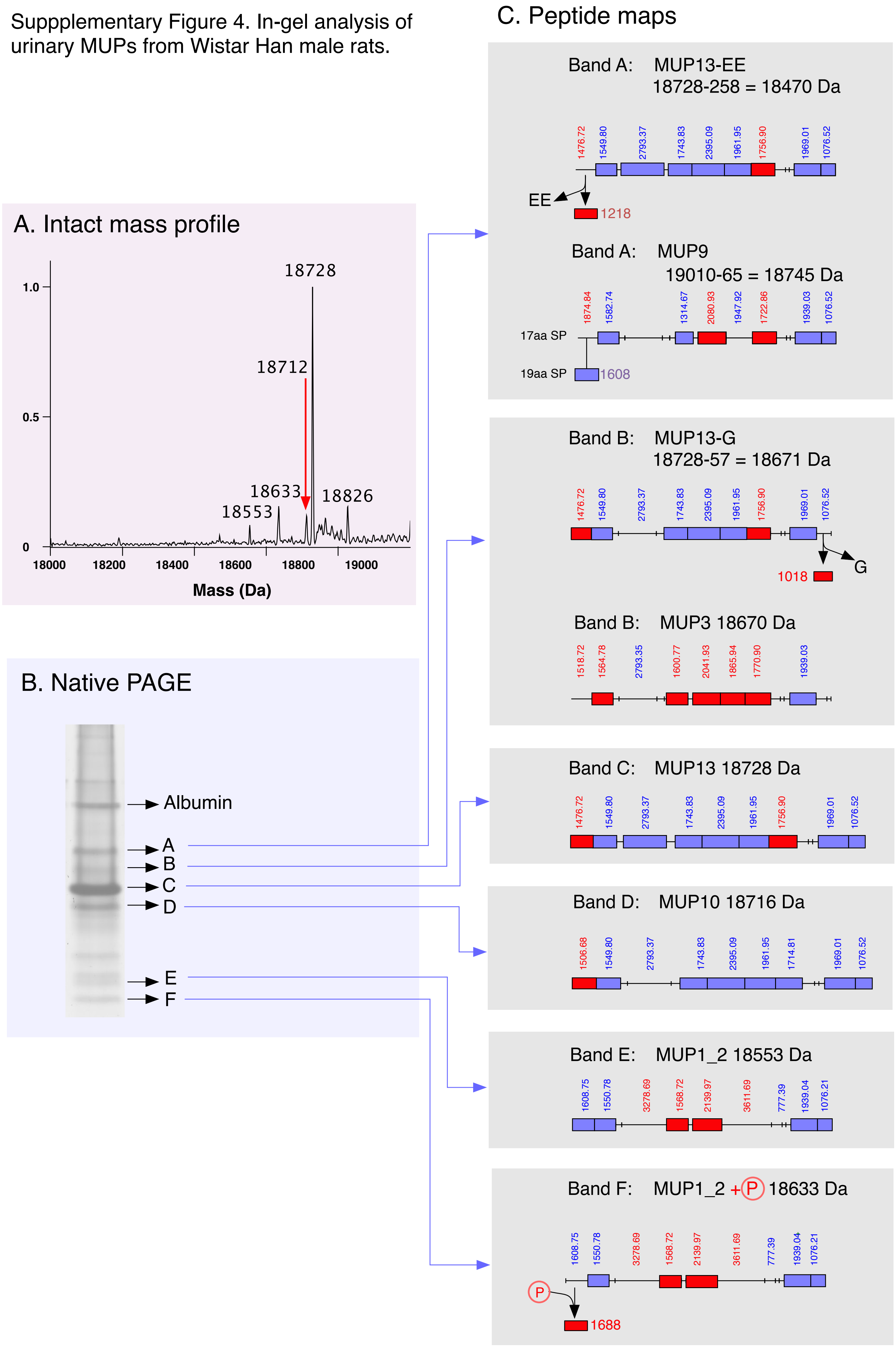

Suppplementary Figure 5. In-gel analysis of urinary MUPs from Brown Norway male rats.

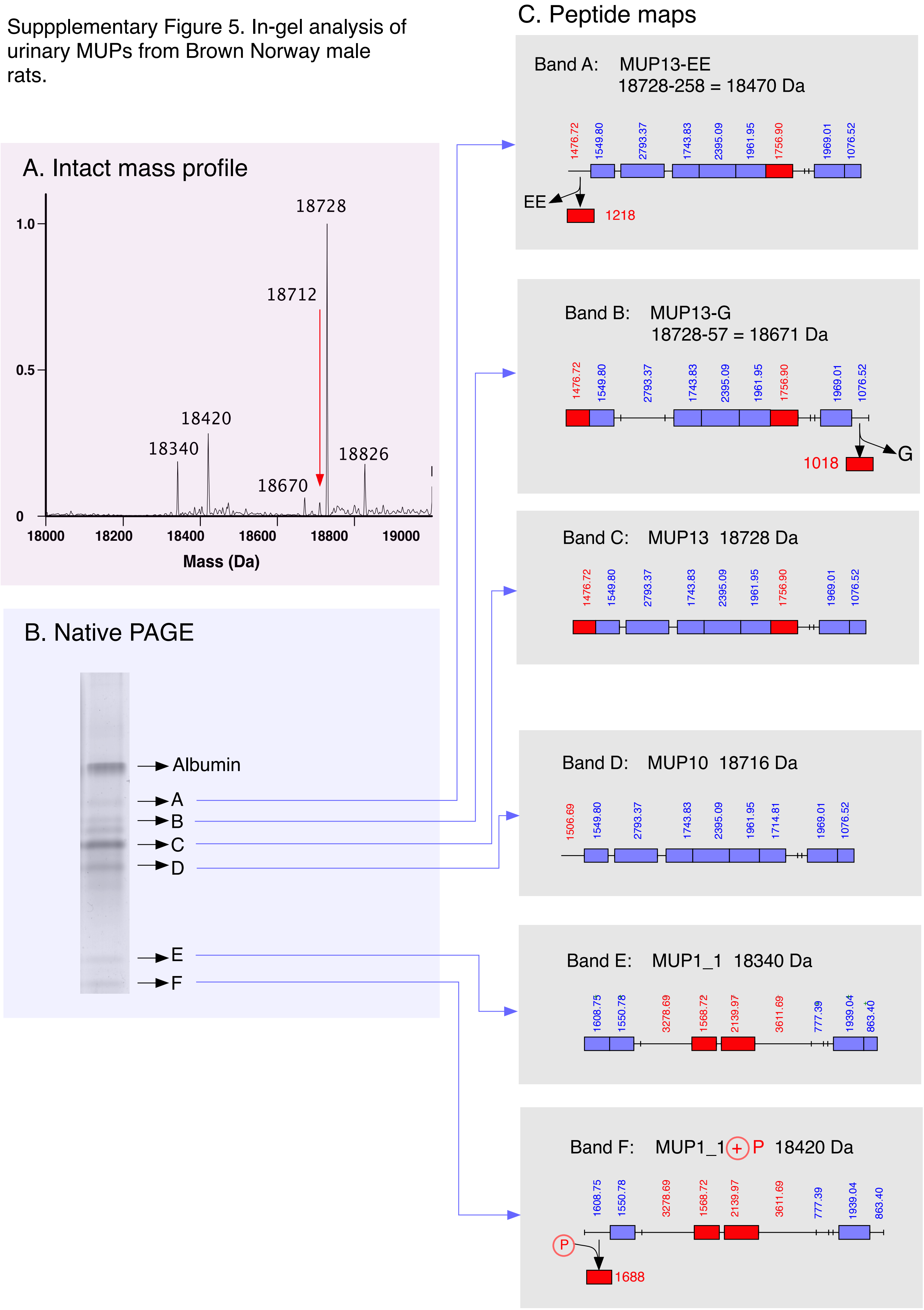

Suppplementary Figure 6. In-gel analysis of urinary MUPs from wild caught male rats.

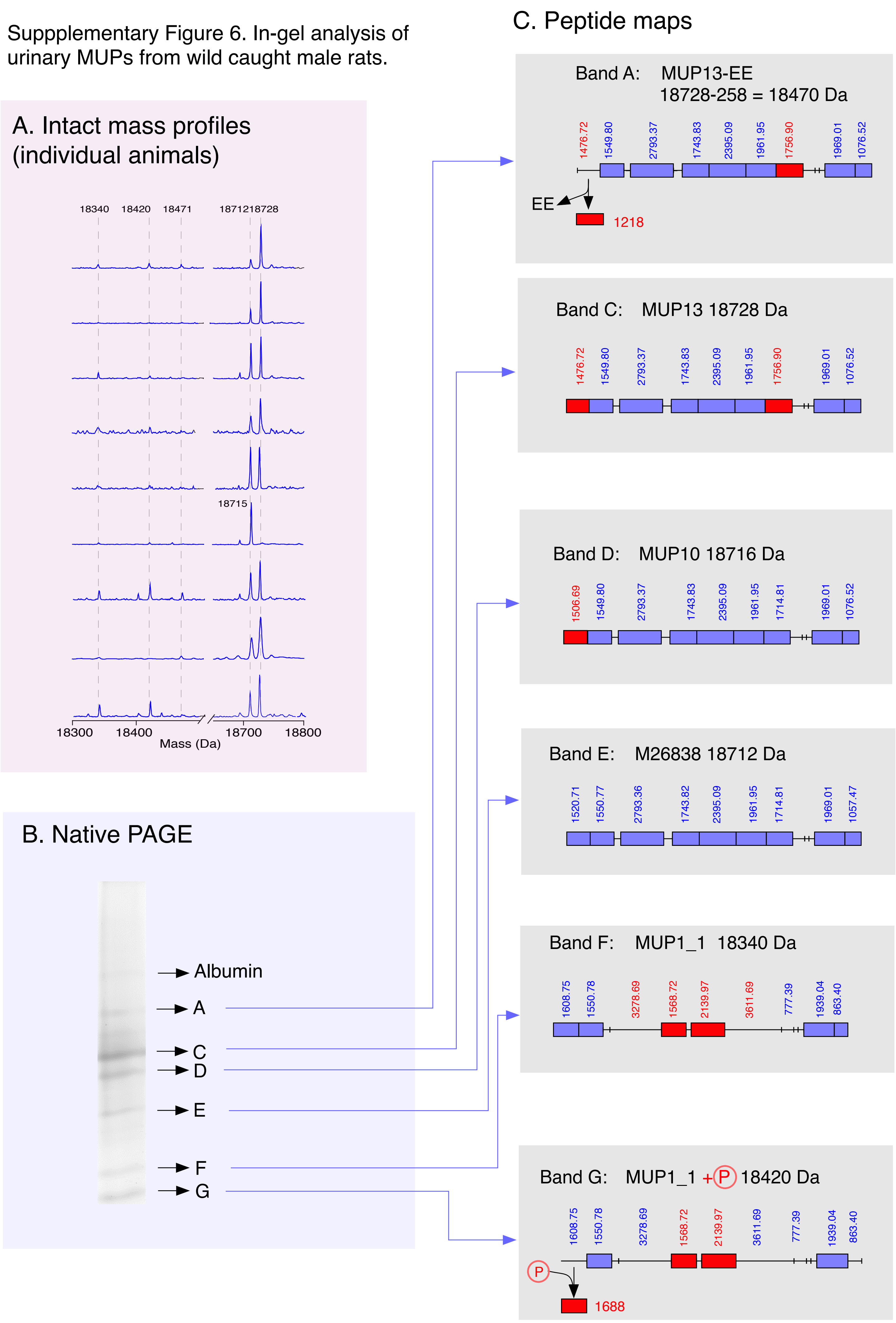

**Supplementary Figure 7: SAX and Intact Mass Profiling from Wistar Han male rats**

## A. SAX Chromatogram

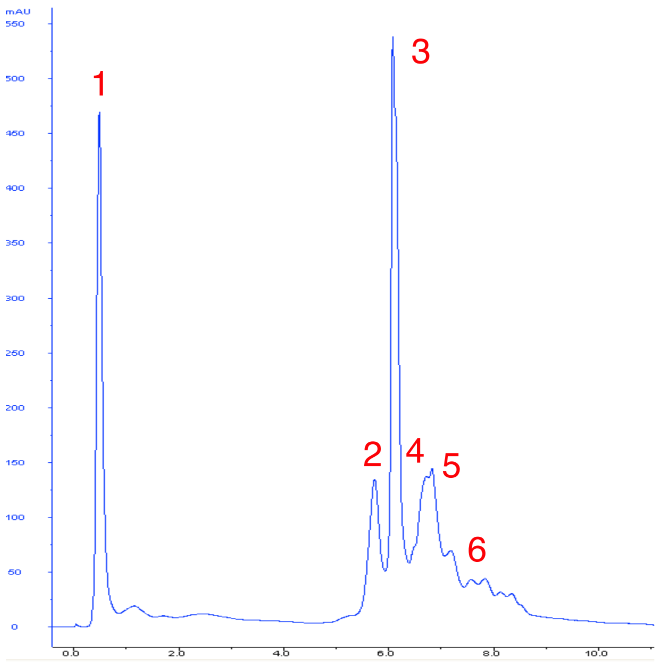


## B. SDS-PAGE of fractions

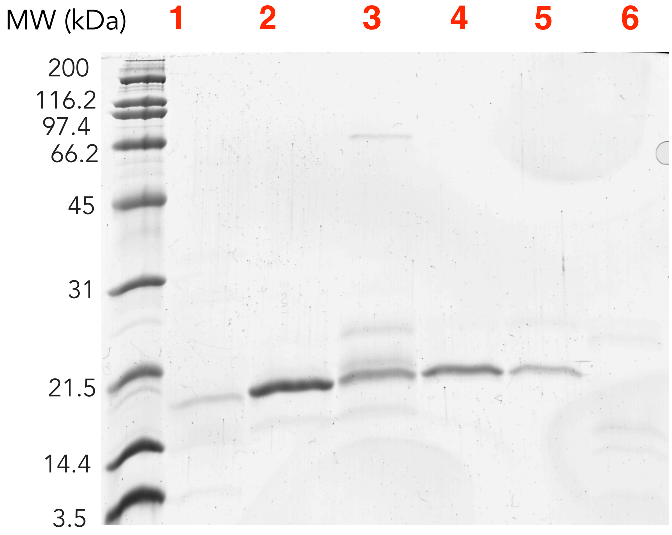


## C. Intact mass profile corresponding to each fraction

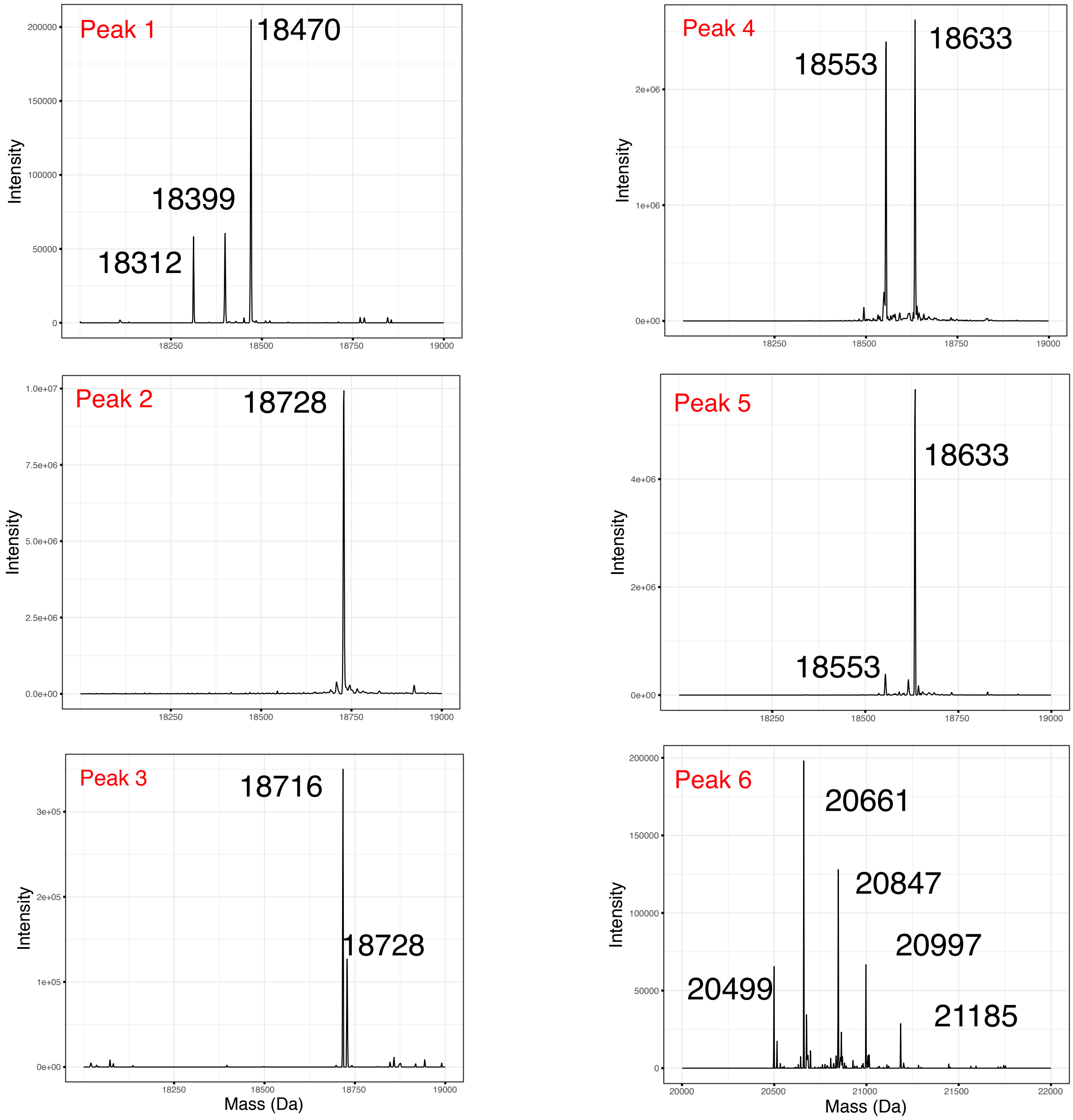

**Supplementary Figure 8: SAX and Intact Mass Profiling from Brown Norway male rats**

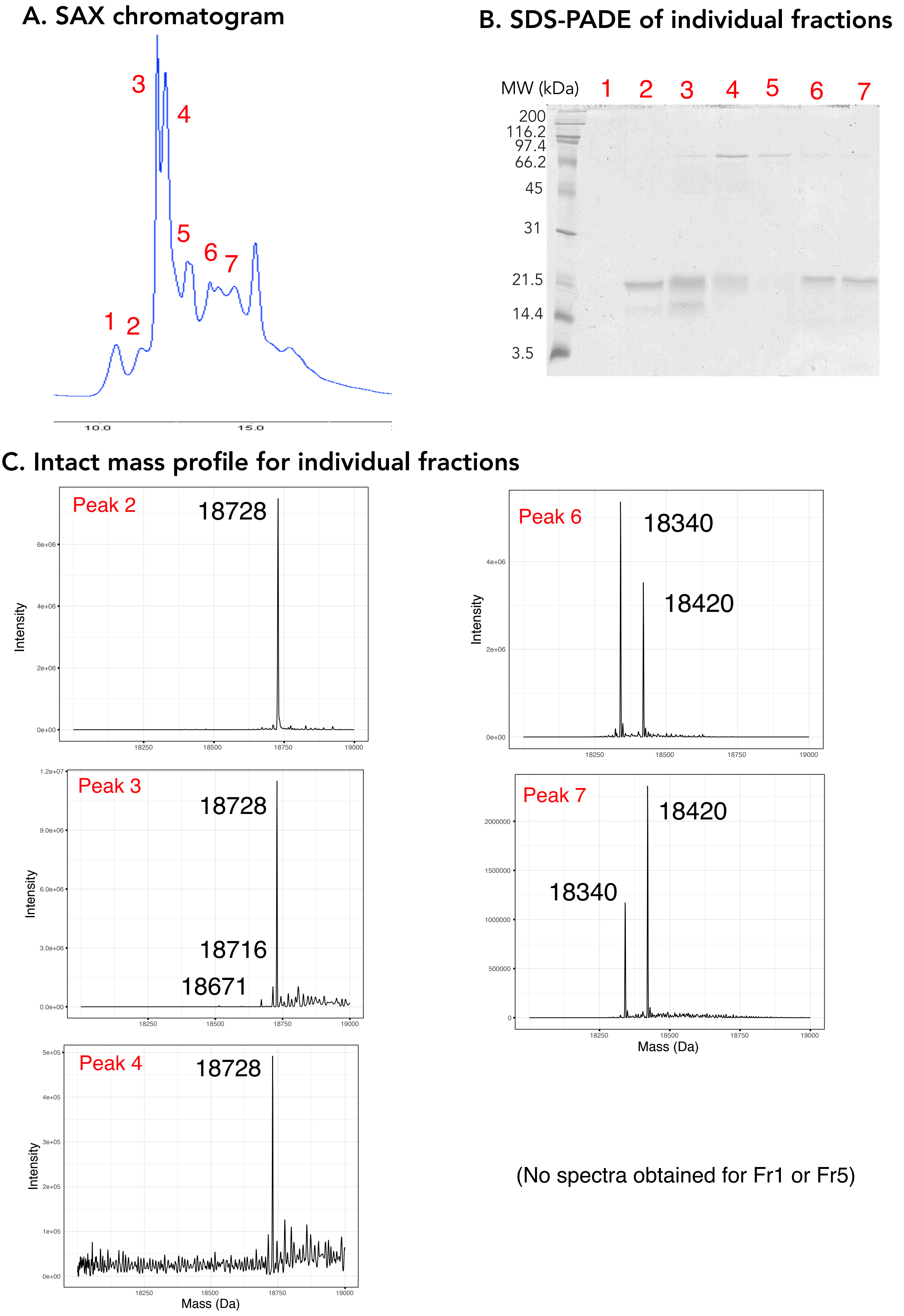

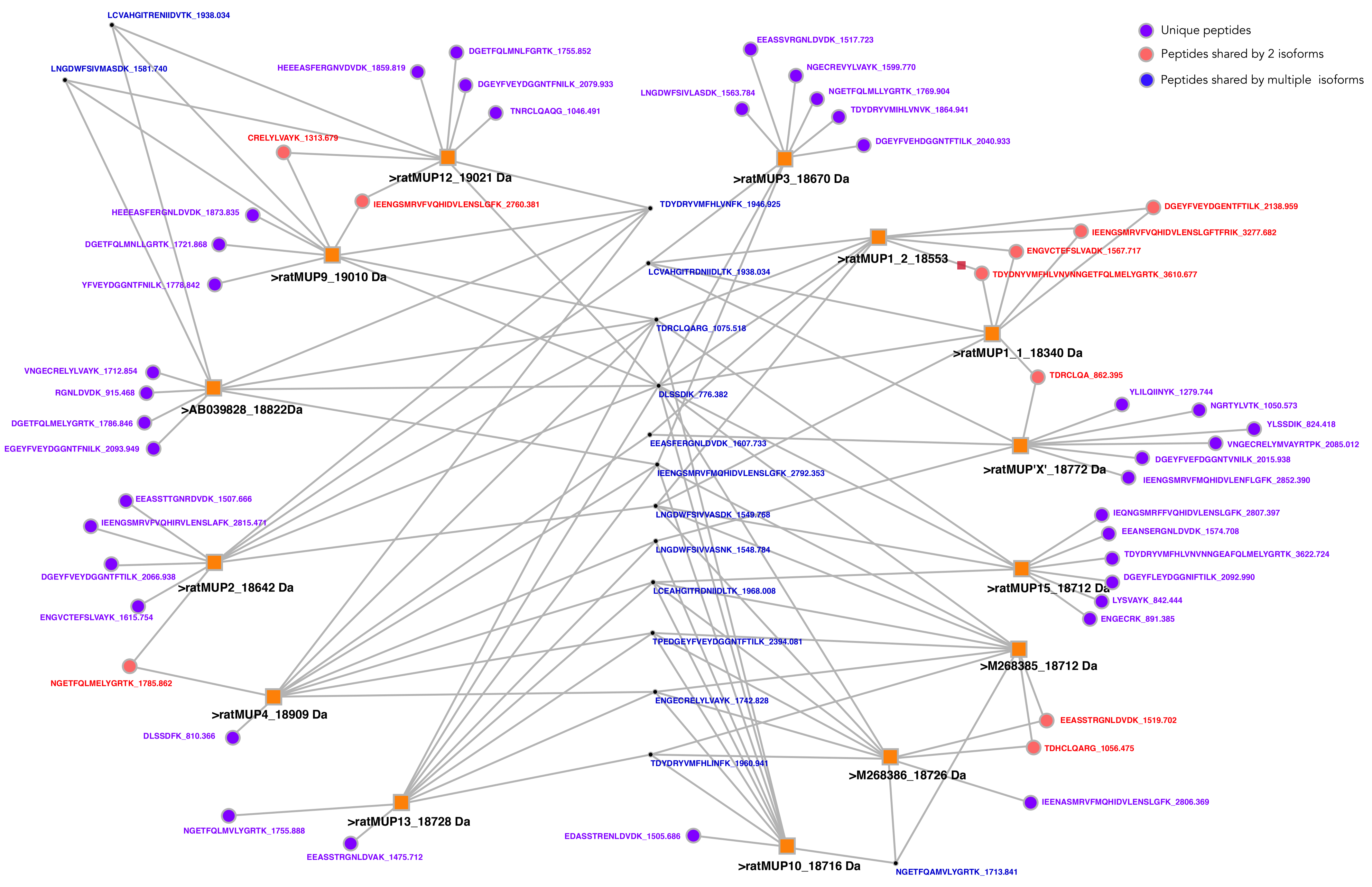

Unique peptides
Peptides shared by 2 isoforms
Peptides shared by multiple isoforms
>ratMUP12_19021 Da
>ratMUP3_18670 Da
>ratMUP9_19010 Da
>ratMUP1_2_18553
>ratMUP1_1_18340 Da
>AB039828_18822Da
>ratMUP'X'_18772 Da
>ratMUP2_18642 Da
>ratMUP15_18712 Da
>M268385_18712 Da
>ratMUP4_18909 Da
>M268386_18726 Da
>ratMUP13_18728 Da
>ratMUP10_18716 Da
LCVAHGITRENIIDVTK_1938.034
LNGDWFSIVMASDK_1581.740
HEEEASFERGNVDVDK_1859.819
DGETFQLMNLFGRTK_1755.852
DGEYFVEYDGGNTFNILK_2079.933
TNRCLQAQG_1046.491
CRELYLVAYK_1313.679
IEENGSMRVFVQHIDVLENSLGFK_2760.381
HEEEASFERGNLDVDK_1873.835
DGETFQLMNLLGRTK_1721.868
YFVEYDGGNTFNILK_1778.842
EEASSVRGNLDVDK_1517.723
NGECREVYLVAYK_1599.770
LNGDWFSIVLASDK_1563.784
NGETFQLMLLYGRTK_1769.904
TDYDRYVMIHLVNVK_1864.941
DGEYFVEHDGGNTFTILK_2040.933
TDYDRYVMFHLVNFK_1946.925
LCVAHGITRDNIIDLTK_1938.034
TDRCLQARG_1075.518
DLSSDIK_776.382
EEASFERGNLDVDK_1607.733
IEENGSMRVFMQHIDVLENSLGFK_2792.353
LNGDWFSIVVASDK_1549.768
LNGDWFSIVVASNK_1548.784
LCEAHGITRDNIIDLTK_1968.008
TPEDGEYFVEYDGGNTFTILK_2394.081
ENGECRELYLVAYK_1742.828
TDYDRYVMFHLINFK_1960.941
NGETFQAMVLYGRTK_1713.841
DGEYFVEYDGENTFTILK_2138.959
IEENGSMRVFVQHIDVLENSLGFTFRIK_3277.682
ENGVCTEFSLVADK_1567.717
TDYDNYVMFHLVNVNNGETFQLMELYGRTK_3610.677
TDRCLQA_862.395
YLILQIINYK_1279.744
NGRTYLVTK_1050.573
YLSSDIK_824.418
VNGECRELYMVAYRTPK_2085.012
DGEYFVEFDGGNTVNILK_2015.938
IEENGSMRVFMQHIDVLENFLGFK_2852.390
VNGECRELYLVAYK_1712.854
RGNLDVDK_915.468
DGETFQLMELYGRTK_1786.846
EGEYFVEYDGGNTFNILK_2093.949
EEASSTTGNRDVDK_1507.666
IEENGSMRVFVQHIRVLENSLAFK_2815.471
DGEYFVEYDGGNTFTILK_2066.938
ENGVCTEFSLVAYK_1615.754
NGETFQLMELYGRTK_1785.862
DLSSDFK_810.366
IEQNGSMRFFVQHIDVLENSLGFK_2807.397
EEANSERGNLDVDK_1574.708
TDYDRYVMFHLVNVNNGEAFQLMELYGRTK_3622.724
DGEYFLEYDGGNIFTILK_2092.990
LYSVAYK_842.444
ENGECRK_891.385
EEASSTRGNLDVDK_1519.702
TDHCLQARG_1056.475
IEENASMRVFMQHIDVLENSLGFK_2806.369
NGETFQLMVLYGRTK_1755.888
EEASSTRGNLDVAK_1475.712
EDASSTRENLDVDK_1505.686

| Wistar Han | | | | | |
|---|---|---|---|---|---|
| BAND | MUP isoform | Peptide sequence | Unique | Theoretical mass (M+H)+ | Observed mass PMF (M+H)+ |
| A | MUP13-EE (18470) | ASSTRGNLDVAK | yes | 1218.62 | 1218.60 ± 0.02 |
| | | LNGDWFSIVVASNK | | 1549.8 | 1549.76 ± 0.03 |
| | | IEENGSMRVFMQHIDVLENSLGFK | | 2793.37 | 2793.30 ± 0.1 |
| | | ENGECRELYLVAYK | | 1743.83 | 1743.81 ± 0.02 |
| | | TPEDGEYFVEYDGGNTFTILK | | 2395.09 | 2395.05 ± 0.02 |
| | | TDYDRYVMFHLINFK | | 1961.95 | 1961.94 ± 0.01 |
| | | NGETFQLMVLYGRTK | yes | 1756.9 | 1756.87 ± 0.02 |
| | | LCEAHGITRDNIIDLTK | | 1969.01 | 1969.00 ± 0.02 |
| | | TDRCLQARG | | 1076.52 | 1076.50 ± 0.02 |
| A | MUP9 (18745 Da) | EEASFERGNLDVDK | | 1608.75 | 1608.78 ± 0.01 |
| | | LNGDWFSIVMASDK | | 1582.75 | 1582.75 ± 0.03 |
| | | CRELYLVAYK | | 1314.68 | 1314.65 ± 0.03 |
| | | YFVEYDGGNTFNILK | yes | 2080.93 | 2080.93 ± 0.03 |
| | | DGETFQLMNLLGRTK | yes | 1722.88 | 1722.85 ± 0.03 |
| | | LCVAHGITRENIIDVTK | | 1939.04 | 1939.05 ± 0.02 |
| | | TDRCLQARG | | 1076.52 | 1076.53 ± 0.01 |
| B | MUP13-G (18671 Da) | EEASSTRGNLDVAK | | 1476.72 | 1476.76 ± 0.03 |
| | | LNGDWFSIVVASNK | | 1549.8 | 1549.80 ± 0.01 |
| | | ENGECRELYLVAYK | | 1743.83 | 1744.82 ± 0.01 |
| | | TPEDGEYFVEYDGGNTFTILK | | 2395.09 | 2395.09 ± 0.01 |
| | | TDYDRYVMFHLINFK | | 1961.95 | 1961.94 ± 0.01 |
| | | NGETFQLMVLYGRTK | | 1756.9 | 1756.91 ± 0.03 |
| | | LCEAHGITRDNIIDLTK | | 1969.01 | 1969.00 ± 0.01 |
| | | TDRCLQAR | | 1019.52 | 1019.54 ± 0.02 |
| B | MUP3 (18670 Da) | LNGDWFSIVLASDK | yes | 1564.78 | 1564.82 ± 0.02 |
| | | NGECREVYLVAYK | yes | 1600.77 | 1600.75 ± 0.02 |
| | | DGEYFVEHDGGNTFTILK | yes | 2041.95 | 2041.93 ± 0.01 |
| | | TDYDRYVMIHLVNVK | yes | 1865.95 | 1865.94 ± 0.02 |
| | | NGETFQLMLLYGRTK | yes | 1770.92 | 1771.93 ± 0.03 |
| | | LCVAHGITRDNIIDLTK | | 1939.04 | 1939.04 ± 0.01 |
| C | MUP13 (18728 Da) | EEASSTRGNLDVAK | yes | 1476.72 | 1476.71 ± 0.02 |
| | | LNGDWFSIVVASNK | | 1549.8 | 1549.80 ± 0.02 |
| | | IEENGSMRVFMQHIDVLENSLGFK | | 2793.37 | 2793.16 ± 0.08 |
| | | ENGECRELYLVAYK | | 1743.83 | 1743.84± 0.02 |
| | | TPEDGEYFVEYDGGNTFTILK | | 2395.09 | 2394.81 ± 0.05 |
| | | TDYDRYVMFHLINFK | | 1961.95 | 1961.94 ± 0.02 |
| | | NGETFQLMVLYGRTK | yes | 1756.9 | 1756.88 ± 0.02 |
| | | LCEAHGITRDNIIDLTK | | 1969.01 | 1969.02 ± 0.02 |
| | | TDRCLQARG | | 1076.52 | 1076.52 ± 0.02 |
| D | MUP10 (18716 Da) | EDASSTRGNLDVDK | yes | 1506.7 | 1506.68 ± 0.02 |
| | | LNGDWFSIVVASNK | | 1549.8 | 1549.78 ± 0.02 |
| | | ENGECRELYLVAYK | | 1743.83 | 1743.84 ± 0.01 |
| | | TPEDGEYFVEYDGGNTFTILK | | 2395.09 | 2395.08 ± 0.03 |
| | | TDYDRYVMFHLINFK | | 1961.95 | 1961.95 ± 0.01 |
| | | NGETFQAMVLYGRTK | | 1714.81 | 1714.83 ± 0.01 |
| | | LCEAHGITRDNIIDLTK | | 1969.01 | 1969.02 ± 0.01 |
| | | TDRCLQARG | | 1076.52 | 1076.53 ± 0.01 |
| E | MUP 1 (18553 Da) | EEASFERGNLDVDK | | 1608.75 | 1608.76 ± 0.02 |
| | | LNGDWFSIVVASDK | | 1550.78 | 1550.76 ± 0.00 |
| | | ENGVCTEFSLVADK | yes | 1568.72 | 1568.73 ± 0.02 |
| | | DGEYFVEYDGENTFTILK | yes | 2139.97 | 2140.00 ± 0.02 |
| | | LCVAHGITRDNIIDLTK | | 1939.04 | 1939.05 ± 0.03 |
| | | TDRCLQARG | | 1076.52 | 1076.53 ± 0.02 |
| F | MUP 1 P (18633 Da) | EEASFERGNLDVDK-P (+80Da) | yes | 1688.75 | 1688.72 ± 0.03 |
| | | LNGDWFSIVVASDK | | 1550.78 | 1550.76 ± 0.01 |
| | | ENGVCTEFSLVADK | yes | 1568.72 | 1568.71 ± 0.01 |
| | | DGEYFVEYDGENTFTILK | yes | 2139.97 | 2139.97 ± 0.03 |
| | | LCVAHGITRDNIIDLTK | | 1939.04 | 1939.03 ± 0.01 |
| | | TDRCLQARG | | 1076.52 | 1076.51 ± 0.01 |

| Brown Norway | | | | | |
|---|---|---|---|---|---|
| BAND | MUP isoform | Peptide sequence | Unique | Theoretical mass (M+H)+ | Observed mass PMF (M+H)+ |
| A | MUP13-EE (18470) | ASSTRGNLDVAK | yes | 1218.62 | 1218.56 ± 0.03 |
| | | LNGDWFSIVVASNK | | 1549.8 | 1549.78 ± 0.02 |
| | | IEENGSMRVFMQHIDVLENSLGFK | | 2793.37 | 2793.40 ± 0.07 |
| | | ENGECRELYLVAYK | | 1743.83 | 1743.82 ± 0.01 |
| | | TPEDGEYFVEYDGGNTFTILK | | 2395.09 | 2395.08 ± 0.01 |
| | | TDYDRYVMFHLINFK | | 1961.95 | 1961.94 ± 0.01 |
| | | NGETFQLMVLYGRTK | yes | 1756.9 | 1756.89 ± 0.02 |
| | | LCEAHGITRDNIIDLTK | | 1969.01 | 1969.02 ± 0.01 |
| | | TDRCLQARG | | 1076.52 | 1076.53 ± 0.01 |
| B | MUP13-G (18671 Da) | EEASSTRGNLDVAK | | 1476.72 | 1476.73 ± 0.01 |
| | | LNGDWFSIVVASNK | | 1549.8 | 1549.79 ± 0.01 |
| | | ENGECRELYLVAYK | | 1743.83 | 1744.83 ± 0.01 |
| | | TPEDGEYFVEYDGGNTFTILK | | 2395.09 | 2395.11 ± 0.01 |
| | | TDYDRYVMFHLINFK | | 1961.95 | 1962.96 ± 0.01 |
| | | NGETFQLMVLYGRTK | | 1756.9 | 1756.88 ± 0.03 |
| | | LCEAHGITRDNIIDLTK | | 1969.01 | 1969.01 ± 0.01 |
| | | TDRCLQAR | | 1019.52 | 1019.52 ± 0.01 |
| C | MUP13 (18728 Da) | EEASSTRGNLDVAK | yes | 1476.72 | 1476.73 ± 0.01 |
| | | LNGDWFSIVVASNK | | 1549.8 | 1549.80 ± 0.01 |
| | | IEENGSMRVFMQHIDVLENSLGFK | | 2793.37 | 2793.38 ± 0.01 |
| | | ENGECRELYLVAYK | | 1743.83 | 1743.82 ± 0.02 |
| | | TPEDGEYFVEYDGGNTFTILK | | 2395.09 | 2395.10 ± 0.02 |
| | | TDYDRYVMFHLINFK | | 1961.95 | 1961.94 ± 0.02 |
| | | NGETFQLMVLYGRTK | yes | 1756.9 | 1756.89 ± 0.01 |
| | | LCEAHGITRDNIIDLTK | | 1969.01 | 1969.02 ± 0.01 |
| | | TDRCLQARG | | 1076.52 | 1076.52 ± 0.01 |
| D | MUP10 (18716 Da) | EDASSTRGNLDVDK | yes | 1506.7 | weak |
| | | LNGDWFSIVVASNK | | 1549.8 | 1549.79 ± 0.01 |
| | | ENGECRELYLVAYK | | 1743.83 | 1743.82 ± 0.01 |
| | | TPEDGEYFVEYDGGNTFTILK | | 2395.09 | 2395.10 ± 0.01 |
| | | TDYDRYVMFHLINFK | | 1961.95 | 1961.95 ± 0.01 |
| | | NGETFQAMVLYGRTK | | 1714.81 | 1714.80 ± 0.01 |
| | | LCEAHGITRDNIIDLTK | | 1969.01 | 1969.02 ± 0.01 |
| | | TDRCLQARG | | 1076.52 | 1076.53 ± 0.01 |
| E | MUP 1 (18553 Da) | EEASFERGNLDVDK | | 1608.75 | 1608.74 ± 0.01 |
| | | LNGDWFSIVVASDK | | 1550.78 | 1550.78 ± 0.01 |
| | | ENGVCTEFSLVADK | yes | 1568.72 | 1568.73 ± 0.01 |
| | | DGEYFVEYDGENTFTILK | yes | 2139.97 | 2139.98 ± 0.02 |
| | | LCVAHGITRDNIIDLTK | | 1939.04 | 1939.05 ± 0.01 |
| | | TDRCLQA | | 863.4 | 863.41 ± 0.01 |
| F | MUP 1 P (18633 Da) | EEASFERGNLDVDK-P (+80Da) | yes | 1688.75 | 1688.73 ± 0.01 |
| | | LNGDWFSIVVASDK | | 1550.78 | 1550.77 ± 0.01 |
| | | ENGVCTEFSLVADK | yes | 1568.72 | 1568.70 ± 0.02 |
| | | DGEYFVEYDGENTFTILK | yes | 2139.97 | 2139.97 ± 0.01 |
| | | LCVAHGITRDNIIDLTK | | 1939.04 | 1939.05 ± 0.01 |

| Wild rats | | | | | |
|---|---|---|---|---|---|
| BAND | MUP isoform | Peptide sequence | Unique | Theoretical mass | Observed mass PMF (M+H)+ |
| A | MUP13-EE (18470) | ASSTRGNLDVAK | yes | 1218.62 | 1218.53;1218.62 |
| | | LNGDWFSIVVASNK | | 1549.8 | 1549.78 ± 0.01 |
| | | IEENGSMRVFMQHIDVLENSLGFK | | 2793.37 | 2793.35 ± 0.09 |
| | | ENGECRELYLVAYK | | 1743.83 | 1743.83 ± 0.01 |
| | | TPEDGEYFVEYDGGNTFTILK | | 2395.09 | 2395.08 ± 0.01 |
| | | TDYDRYVMFHLINFK | | 1961.95 | 1961.94 ± 0.01 |
| | | NGETFQLMVLYGRTK | yes | 1756.9 | 1756.90 ± 0.02 |
| | | LCEAHGITRDNIIDLTK | | 1969.01 | 1969.00;1969.01 |
| | | TDRCLQARG | | 1076.52 | 1076.54 ± 0.01 |
| C | MUP13 (18728 Da) | EEASSTRGNLDVAK | yes | 1476.72 | 1476.72 ± 0.01 |
| | | LNGDWFSIVVASNK | | 1549.8 | 1549.80 ± 0.01 |
| | | IEENGSMRVFMQHIDVLENSLGFK | | 2793.37 | 2793.42 ± 0.03 |
| | | ENGECRELYLVAYK | | 1743.83 | 1743.83 ± 0.01 |
| | | TPEDGEYFVEYDGGNTFTILK | | 2395.09 | 2395.08 ± 0.02 |
| | | TDYDRYVMFHLINFK | | 1961.95 | 1961.95 ± 0.01 |
| | | NGETFQLMVLYGRTK | yes | 1756.9 | 1756.89 ± 0.01 |
| | | LCEAHGITRDNIIDLTK | | 1969.01 | 1969.02 ± 0.01 |
| | | TDRCLQARG | | 1076.52 | 1076.53 ± 0.01 |
| D | MUP10 (18716 Da) | EDASSTRGNLDVDK | yes | 1506.7 | 1506.8 ± 0.01 |
| | | LNGDWFSIVVASNK | | 1549.8 | 1549.79 ± 0.02 |
| | | IEENGSMRVFMQHIDVLENSLGFK | | 2793.37 | 2793.40 ± 0.06 |
| | | ENGECRELYLVAYK | | 1743.83 | 1743.82 ± 0.02 |
| | | TPEDGEYFVEYDGGNTFTILK | | 2395.09 | 2395.10 ± 0.01 |
| | | TDYDRYVMFHLINFK | | 1961.95 | 1961.96 ± 0.01 |
| | | NGETFQAMVLYGRTK | | 1714.81 | 1714.83 ± 0.02 |
| | | LCEAHGITRDNIIDLTK | | 1969.01 | 1969.02 ± 0.01 |
| | | TDRCLQARG | | 1076.52 | 1076.52 ± 0.01 |
| E | M26838 (18712 Da) | EEASSTRGNLDVDK | | 1520.72 | 1520.71 ± 0.01 |
| | | LNGDWFSIVVASDK | | 1550.78 | 1550.77 ± 0.01 |
| | | IEENGSMRVFMQHIDVLENSLGFK | | 2793.37 | 2793.40 ± 0.02 |
| | | ENGECRELYLVAYK | | 1743.84 | 1743.83 ± 0.01 |
| | | TPEDGEYFVEYDGGNTFTILK | | 2395.09 | 2395.09 ± 0.01 |
| | | TDYDRYVMFHLINFK | | 1961.95 | 1961.96 ± 0.01 |
| | | NGETFQAMVLYGRTK | | 1714.81 | 1714.86 ± 0.02 |
| | | LCEAHGITRDNIIDLTK | | 1969.01 | 1969.01 ± 0.01 |
| | | TDHCLQARG | | 1057.48 | 1057.48 ± 0.01 |
| F | MUP 1 (18553 Da) | EEASFERGNLDVDK | | 1608.75 | 1608.76 ± 0.01 |
| | | LNGDWFSIVVASDK | | 1550.78 | 1550.78 ± 0.01 |
| | | ENGVCTEFSLVADK | yes | 1568.72 | 1568.74 ± 0.01 |
| | | DGEYFVEYDGENTFTILK | yes | 2139.97 | 2139.98 ± 0.01 |
| | | LCVAHGITRDNIIDLTK | | 1939.04 | 1939.05 ± 0.01 |
| | | TDRCLQA | | 863.4 | 863.42 ± 0.01 |
| G | MUP 1 P (18633 Da) | EEASFERGNLDVDK-P (+80Da) | yes | 1688.75 | 1688.71 ± 0.02 |
| | | LNGDWFSIVVASDK | | 1550.78 | 1550.77 ± 0.01 |
| | | ENGVCTEFSLVADK | yes | 1568.72 | 1568.70 ± 0.02 |
| | | DGEYFVEYDGENTFTILK | yes | 2139.97 | 2139.98 ± 0.01 |
| | | LCVAHGITRDNIIDLTK | | 1939.04 | 1939.05 ± 0.01 |
